# Repetitive Mild Traumatic Brain Injury in Rats Impairs Cognition, Enhances Prefrontal Cortex Neuronal Activity, and Reduces Pre-synaptic Mitochondrial Function

**DOI:** 10.3389/fncel.2021.689334

**Published:** 2021-08-10

**Authors:** Yin Feng, Keguo Li, Elizabeth Roth, Dongman Chao, Christina M. Mecca, Quinn H. Hogan, Christopher Pawela, Wai-Meng Kwok, Amadou K. S. Camara, Bin Pan

**Affiliations:** ^1^Department of Anesthesiology, Medical College of Wisconsin, Milwaukee, WI, United States; ^2^Department of Cell Biology, Neurobiology and Anatomy, Medical College of Wisconsin, Milwaukee, WI, United States; ^3^Department of Biomedical Engineering, Medical College of Wisconsin, Milwaukee, WI, United States; ^4^Department of Biophysics, Medical College of Wisconsin, Milwaukee, WI, United States; ^5^Department of Pharmacology and Toxicology, Medical College of Wisconsin, Milwaukee, WI, United States; ^6^Department of Physiology, Medical College of Wisconsin, Milwaukee, WI, United States

**Keywords:** mild traumatic brain injury, rat model, cognitive impairment, neuronal activity, mitochondrial function

## Abstract

A major hurdle preventing effective interventions for patients with mild traumatic brain injury (mTBI) is the lack of known mechanisms for the long-term cognitive impairment that follows mTBI. The closed head impact model of repeated engineered rotational acceleration (rCHIMERA), a non-surgical animal model of repeated mTBI (rmTBI), mimics key features of rmTBI in humans. Using the rCHIMERA in rats, this study was designed to characterize rmTBI-induced behavioral disruption, underlying electrophysiological changes in the medial prefrontal cortex (mPFC), and associated mitochondrial dysfunction. Rats received 6 closed-head impacts over 2 days at 2 Joules of energy. Behavioral testing included automated analysis of behavior in open field and home-cage environments, rotarod test for motor skills, novel object recognition, and fear conditioning. Following rmTBI, rats spent less time grooming and less time in the center of the open field arena. Rats in their home cage had reduced inactivity time 1 week after mTBI and increased exploration time 1 month after injury. Impaired associative fear learning and memory in fear conditioning test, and reduced short-term memory in novel object recognition test were found 4 weeks after rmTBI. Single-unit *in vivo* recordings showed increased neuronal activity in the mPFC after rmTBI, partially attributable to neuronal disinhibition from reduced inhibitory synaptic transmission, possibly secondary to impaired mitochondrial function. These findings help validate this rat rmTBI model as replicating clinical features, and point to impaired mitochondrial functions after injury as causing imbalanced synaptic transmission and consequent impaired long-term cognitive dysfunction.

## Introduction

According to the United States Center for Disease Control and Prevention, there are 2.87 million traumatic brain injury (TBI) -related emergency department visits, hospitalizations, and deaths combined per year in the United States. Over 75% of TBIs are mild (mTBI) or concussions. mTBI is an established risk factor for the development of chronic traumatic encephalopathy, post-traumatic stress disorder, neurodegenerative diseases, and neuropsychiatric disorders many years after the initial injury (Cole and Bailie, 2016). In humans, multi-injuries occur frequently with short injury intervals, for example a football player may experience a helmet-to-helmet hit before hitting the ground, or a driver in an automobile accident may experience multiple hits from the airbag and the seat’s headrest. Patients with a history of multiple concussions are also at higher risk for prolonged symptoms than after a single injury (Eisenberg et al., 2013). However, most animal studies to date modeled single mTBI (Hoogenboom et al., 2019). Repeated mTBI (rmTBI) preclinical models mimicking those scenarios in humans are needed.

The acute response of brain tissue to a mechanical insult results in ongoing pathology and neurologic outcomes that appear weeks or months following the initial injury (Bramlett and Dietrich, 2015), including diffuse axonal injury (Johnson et al., 2013) and accumulation of amyloid precursor protein (Collins et al., 2020). Furthermore, multiple reports identify mitochondrial dysfunction following mTBI (Vagnozzi et al., 2007; Johnson et al., 2013; Fischer et al., 2016; Kim et al., 2017). However, despite these general findings, specific cellular mechanisms underlying rmTBI remain undefined.

A major hurdle for establishing clinical rmTBI pathophysiology is the uncertain validity of animal models used to replicate rmTBI, which limits the development of new therapeutic approaches (Xiong et al., 2013; Wojnarowicz et al., 2017). The repeated closed-head impact model of engineered rotational acceleration model (rCHIMERA) was recently developed for mice (Namjoshi et al., 2014; Namjoshi et al., 2017) and rats (Vonder Haar et al., 2019). This rmTBI model reliably produces the expected histological changes, including diffuse axonal injury, reactive microgliosis, release of inflammatory cytokines, and hyperphosphorylated tau proteinopathy (Namjoshi et al., 2014; Vonder Haar et al., 2019). These findings indicate it may be a reliable and potentially good alternative to existing surgical injury models.

Another challenge in preclinical studies is the inherently high variance of the behavioral assays typically used to detect changes after rmTBI. Neurotrauma studies using the traditional approaches of the elevated plus maze, rotarod, passive avoidance, radial arm water maze, novel object recognition, fear conditioning, Morris water maze, and Barnes maze to test spatial memory, anxiety, and motor functions (Bodnar et al., 2019) have generated inconsistent findings within the same injury model or between different injury models (Bodnar et al., 2019). To remove the putative impact of differences in behavioral setups, animal handling and/or testing procedures, we developed an approach for evaluating behaviors with limited stress. We achieved this objective by employing automated analyses of spontaneous activities in the open field home-cage environment.

Since astrocytes and microglia activation are considered key players in initiating inflammatory response after injury and are biomarkers for TBI (Hernandez-Ontiveros et al., 2013; Corps et al., 2015; Karve et al., 2016; Pham et al., 2019; Di Battista et al., 2020), we detected the expression of markers of astrogliosis and microgliosis in different brain areas at multiple time points. As abnormal cortical neuronal activity is a key pathophysiological feature of rmTBI (Goforth et al., 2011; Johnstone et al., 2014), we evaluated neuronal activity to validate this rmTBI model. Furthermore, we explored the synaptic mechanisms of rmTBI-induced abnormal neuronal activity, including effects of rmTBI on mitochondrial regulation of synaptic function in the mPFC, a key area for cognitive function (Euston et al., 2012).

## Materials and Methods

### Animals

Male Sprague-Dawley rats weighing 280–300 g were obtained from the Envigo Corporation (Indianapolis, IN, United States), and were maintained and used according to the National Institutes of Health Guide for the Care and Use of Laboratory Animals. All rat experiments were performed according to protocols approved by the Institutional Animal Care and Use Committee of the Medical College of Wisconsin. Animals were housed in a pathogen-free facility, 2 animals per ventilated cage, in a room maintained at 25 ± 1°C at 35 to 45% humidity, with a 12/12-h day/night cycle. Animals had free access to food and water. At the termination of each study, euthanasia was performed by decapitation during deep isoflurane anesthesia.

### rmTBI

A surgery-free rmTBI approach using the rCHIMERA (device was obtained from the University of British Columbia, Canada) was performed with modifications (Namjoshi et al., 2014; Vonder Haar et al., 2019). Briefly, rats were sedated with dexmedetomidine to avoid possible effects of commonly used volatile anesthetics on cognitive, motor, and histological outcomes after neurological injury (Rowe et al., 2013; Slupe and Kirsch, 2018) and mitochondrial functions (Ljubkovic et al., 2007; Zhang et al., 2012). Dexmedetomidine (0.2 mg/kg/hour, Sigma-Aldrich, St. Louis, MO, United States) was administered by tail vein injection for 15–20 min before the head impact. This induced inactivity but rats retained a withdrawal response to noxious paw pinch by a toothed forceps and blink reflex to corneal stimulation by a cotton wisp. After confirming loss of withdrawal from innocuous paw stimulation with fingers and loss of righting reflex, rats were placed supine in the holding bed and secured firmly to the device with a strap around the abdomen. Another strap was loosely fastened around the thorax to prevent hyperflexion of the thoracic spine in the sagittal plane during impact. The top of the animal’s head lay flat over a hole in the head plate so that the piston can strike the vertex of the head covering a 5 mm^2^ area around bregma. Piston velocities were obtained using photogate sensors on the rat rCHIMERA device. Three impacts were delivered each day at 6.4 m/s (2 J) with 1 min interval between them. Immediately after the impacts, the dexmedetomidine infusion was discontinued, animals were injected with one dose of the dexmedetomidine antidote atipamezole hydrochloride (Revertidine, Modern Veterinary Therapeutics, Miami, FL, United States; Intraperitoneal injection, 1.5 mg/kg), and placed in a heated recovery cage until full motor function recovered. Another group of three impacts were delivered 48 h later. This injury protocol with the three repeated impacts with 1 min interval on the first day and repeated 48 h later was chosen to mimic the scenarios that athletes and veterans are likely exposed to repeated head impacts even without experiencing concussive symptoms in a short period of time (McKee and Robinson, 2014; Davenport et al., 2016; Nolan et al., 2018; Hoogenboom et al., 2019). Sham injury rats underwent the same procedures except for the impacts.

#### Neurological Severity Evaluation

Neurological severity was assessed by using the modified Neurological Severity Score (mNSS) (Yarnell et al., 2016) determined at 1, 2, and 7 days following the last impact. mNSS is a composite of 10 different tasks including general balance test, landing test, tail raise test, drag test, righting reflex, ear reflex, eye reflex, sound reflex, tail reflex, and paw flexion, which assess motor function, alertness, and physiological behavior (Yarnell et al., 2016).

#### Spontaneous Behavioral Tests

##### Open field

Behavior of a rat in a new environment (an open field) contains sufficient complexity and sensitivity to a wide range of neurological disorders. The Behavioral Spectrometer is a newly developed device that is capable of automatically identifying 23 unique behaviors and providing a complete, real-time profile of animal behavior in an open field scenario (Brodkin et al., 2014). The apparatus (Biobserve Inc., Bonn, Germany) consists of a 40 cm by 40 cm square area enclosed in a cube box with edge length of 45 cm. A camera is mounted in the ceiling above the arena to monitor animal’s position and posture. A row of 32 infrared transmitter and receiver pairs is embedded in the walls to monitor the rat behaviors. Accelerometers embedded in the floor are used to capture the rat’s vibrations for detecting the rearing behaviors. Combination of these sensors and detailed descriptions of rat behavior can be generated. Each behavior produces a distinct pattern of sensor data, such that 23 different patterns of sensor readings are detected and recorded by the software (Viewer3, Biobserve).

##### Home cage

Spontaneous activities such as locomotor activity, feeding, rearing, hanging, and sleeping of rats in a familiar environment (their home cages) were recorded and analyzed with a CleverSys HomeCageScan system (CleverSys Inc., Reston, VA, United States). The rats remained in the cages in which they had resided for 24 h prior to the recording interval such that they were familiar with the environment, and their activities were recorded for the subsequent 24 h (12 h nighttime and 12 h daytime). During the recording time, the experimenters were not present in the isolated housing area and monitored the status of the rats remotely, to minimize interaction and reduce stress. The data from home cages were grouped into the following activities: exploration (dig, forage, and sniff), inactivity (remain low, awaken, twitch, sleep, pause, and stationary), feeding (eat, drink, and chew), walking (walk left, walk right, walk slowly, and circle), traveling (total length traveled), vertical (rear up, come down from partially reared, come down to partially reared from fully reared up position, rear up full from partial, rear up partially, remain rear up, remain partially reared, jump, repetitive jump, and come down), and miscellaneous (turn, stretch body, unknown behavior, urinate, and groom).

#### Rotarod

The motor coordination and motor learning of rats was evaluated with a rotarod apparatus (IITC Life Science, Woodland Hills, CA, United States). The procedure included 3 days training and 1 day testing. Rats in their home cages were placed in the testing room for at least 1 h before testing to minimize effects of stress. Rats were trained to be able to walk forward while maintaining their balance on the rod at a constant speed of 5 rotations per minute (RPM). Three 60 s-trials with 10 min inter-trial intervals each day were conducted for three consecutive days. On testing day, the apparatus was set to accelerate from 5 to 40 RMP in 300 s and each trial ended when rats fell off the rod. This procedure was repeated three times separated by 15 min inter-trial intervals. Latency to fall and the speed of falling were recorded for analysis.

#### Novel Object Recognition

Visual/recognition memory was assessed with the novel object recognition (NOR) test (Barker et al., 2007; Pan et al., 2011a), using the Behavioral Spectrometer apparatus. Initially, rats were habituated in the box for 15 min daily for 2 days to reduce stress due to the new environment. After 24 h of habituation, the novel object preference task was performed.

##### Novel object preference task

The procedure comprised an acquisition (training) phase, followed by a preference test after a delay of 5 min. In the acquisition phase, duplicate objects (assemblies made from Lego interlocking plastic bricks that differed in shape, color, and size, and were heavy enough to prevent the rat from moving them) were placed near the two corners at either end of one side of the arena. The animal was placed into the arena for a total of 5 min, and proximity to the objects measured by time spent in that quadrant of the arena was recorded. Rats that showed a clear preference (>60%) for one object during the acquisition phase were excluded from the experiment. Five minutes after the acquisition phase, the rat was again placed in the middle of the arena and presented with two objects in the same positions, one of which was a new object identical to the ones used in the acquisition phase, while the other object was novel in design. The positions of the objects in the test and the objects used as novel or familiar were counterbalanced between the rats. Recording of the rat exploring the chamber and objects was continued for 5 min. Analysis of the recorded videos used automated software (EthoVision XT, Noldus Information Technology Inc., Leesburg, VA, United States) to determine the time spent exploring each object. The exploration behavior was defined as directing the head toward the object 2 cm or less. Walking around or sitting on the object was not considered as exploratory behavior. Discrimination index was calculated as the difference in time spent by each rat on novel and familiar objects divided by the total time spent exploring both objects (Sivakumaran et al., 2018). Data obtained in the first 2 min are presented.

#### Fear Conditioning

Using an auditory fear conditioning paradigm, contextual/cued fear conditioning and trace fear conditioning were measured.

##### Contextual/cued fear conditioning

On day 1, rats were placed individually into a standard conditioning chamber (Coulbourn Instruments, Holliston, MA, United States) within a sound- and light-attenuating box illuminated by one 25 W bulb. The experimental contingencies were controlled by a computer via FreezeFrame software from Coulbourn. Rats were placed in the chamber for 3 min (baseline) before presenting a 20-s, 80-dB, and 3-kHz tone, conditioned stimulus (CS). During the last 2 s of the tone, an unconditioned stimulus (US) consisting of a 0.5 mA foot shock was delivered through the grid floor. The pairing of CS and US was repeated seven times at intervals of 2 min to strengthen the association between the tone and the shock. Memory was assessed 24 h later in a novel context that differed in scent, color/texture of walls and floor, and illumination, by measuring the amount of time rats exhibited freezing in response to the tone. Following this initial determination, extinction was tested by recording freezing behavior during the presentation of 10 tones (separated by 200 s) without a foot shock every 24 h after conditioning for 5 consecutive days.

##### Trace fear conditioning

Trace fear conditioning is like cued fear conditioning except the CS (0.5 mA foot aversive shock) is presented 60 s after the US terminated. For context testing, animals were put in the same environment for conditioning without US and CS, and freezing time was recorded. For trace test, rats were put into a new environment and given CS (tone), and freezing time was recorded for a period of 5 min.

### Immunohistochemistry

Immunohistochemical procedures were based on our previous study with minor modifications (Pan et al., 2011b). Rats were anesthetized with 3–4% isoflurane and perfused through the aorta with 4% paraformaldehyde in 0.1 M sodium PBS with 4% sucrose, pH 7.4. After perfusion, brains were removed and fixed in the same fixative overnight at 4°C. The brains were then cryoprotected in increasing concentrations of sucrose (10, 20, and 30%) in 0.1 M phosphate-buffered saline (PBS) at 4°C, frozen on dry ice, and stored at −80°C until use. Coronal sections were cut at 30 μm thickness with a cryostat. After H_2_O_2_ treatment and rinsing three times in PBS, free floating sections were blocked for 1 h at room temperature with blocking solution (1% bovine serum albumin, 5% normal goat serum, and 1% Triton X-100 in 0.1 M PBS, pH 7.4). Sections were then incubated with 1:350 GFAP (glial fibrillary acidic protein, a biomarker for assessing astrogliosis/astrocyte proliferation) antibody (anti-GFAP; Cell Signaling Technology) and 1:300 of Iba-1 (ionized calcium binding adaptor molecule 1, a microglial/macrophage marker) antibody (anti-Iba-1; Cell Signaling Technology) at 4°C for 24 h. After rinsing three times, 5 min each, in PBS, sections were incubated with the secondary antibodies: 1:100 goat anti-rabbit IgG-HRP (Bio-Rad) for 2 h at room temperature. Control sections were processed with a non-immune serum in place of the primary antibodies. Sections were analyzed by using a fluorescence microscope (BZ-X800, Keyence Corporation of United States, Itasca, IL, United States). For each rat, three sections (20 μm thick) of mPFC, dentate gyrus, and corpus callosum with 200 μm space were cut and examined; the mean counts of GFAP or Iba-1-positive microglia/macrophages were determined manually. Averaged areas of GFAP and Iba-1 positive cells were calculated with ImageJ^1^ by determining the total positive areas in each area divided by the number of positive cells, which represents morphological changes of glia cells after injury.

### *In vivo* Electrophysiology

A craniotomy was performed and the dura opened to expose the mPFC. The brain surface was covered in warm mineral oil and a single barreled glass micropipette filled with solution containing 1 M NaCl (with resistance of 15–20 MΩ) was advanced into the mPFC using a microdrive (David Kopf Instruments, Tujunga, CA, United States) at 2 μm per step, targeting layer IV/V at depths of 3.4–4.2 mm from the brain surface using the stereotaxic coordinates of 3.0–3.4 mm anterior to bregma, 0.7 mm lateral to midline (Paxinos and Watson, 2014). For each rat, spontaneous neuronal activity (SA) was sought by the following process. Four vertical pathways with 100 μm interval from bregma +3.0 to +3.4 were examined. At each pathway, SA of pyramidal neurons was sought within a 30 μm span starting from 3.4 mm from the brain surface by increments of 1 μm, after which the electrode was advanced 50 μm to initiate a subsequent 30 μm search. This process was repeated two more times before moving to the next pathway. In this manner, 16 mPFC subregions were examined on one side (chosen randomly) of each animal, and the incidence of SA was calculated as the percentage of area with SA. mPFC pyramidal neurons can be distinguished from interneurons based on their broader action potential waveform and lower baseline discharge rate (Ji and Neugebauer, 2011). Signals were collected with an Axoclamp 900A microelectrode amplifier (Molecular Devices, San Jose, CA, United States), filtered at 1 kHz, and sampled at 10 kHz using a digitizer (DigiData 1440 A). Action potentials were isolated by setting the threshold above background noise, and individual units were identified by template matching using Spike2 (Cambridge Electronic Design Limited, Cambridge, United Kingdom) or pClamp 11 (Molecular Devices).

### Slice Preparation and Electrophysiology

Rats were anesthetized by isoflurane inhalation and decapitated. mPFC slices (250 μm) and whole-cell recordings were made as described previously (Pan et al., 2011b). Slices were stored in artificial cerebrospinal fluid (aCSF) containing (in mM): 119 NaCl, 2.5 KCl, 2.5 CaCl_2_, 1 MgCl_2_, 1.25 NaH_2_PO_4_, 26 NaHCO_3_, and 10 glucose at room temperature. All solutions were saturated with 95% O_2_ and 5% CO_2_. All recordings were performed at 32 ± 1°C by using an automatic temperature controller (Warner Instrument, Hamden, CT, United States). Patch pipettes, ranging from 2–4 MΩ resistance, were formed from borosilicate glass (King Precision Glass Co., Claremont, CA, United States) and fire polished. Recordings were made with an Axopatch 700B amplifier (Molecular Devices, Downingtown, PA, United States). Miniature excitatory postsynaptic currents (mEPSCs) were recorded from mPFC pyramidal neurons. Gamma-Aminobutyric acid receptor type A (GABAA) blocker picrotoxin (50 μM) was present in the aCSF throughout the experiments. Glass pipettes (3–5 MΩ) were filled with an internal solution containing (in mM): 130 cesium methanesulfonate, 10 CsCl, 5 QX-314, 10 HEPES, 0.2 EGTA, 2 MgCl_2_, 4 MgATP, 0.3 Na_2_GTP, and 10 Na_2_-phosphocreatine (pH 7.2 with CsOH). Miniature inhibitory postsynaptic currents (mIPSCs) were recorded the same pattern as for mEPSCs. Glutamate receptor antagonists 6-cyano-7-nitroquinoxaline-2,3-dione disodium (CNQX, 20 μM) and D-2-amino-5-phosphonovaleric acid (D-AP-5, 50 μM) were present in the aCSF. The internal solution in patch pipettes contained (in mM): 80 Cs-methanesulfonate, 60 CsCl, 2 QX-314, 10 HEPES, 0.2 EGTA, 2 MgCl_2_, 4 MgATP, 0.3 Na_2_GTP, and 10 Na_2_-phosphocreatine (pH 7.2 with CsOH). For both miniature EPSCs (mEPSCs) and IPSCs (mIPSCs), tetrodotoxin (TTX) was added in the aCSF to block action potentials. To ascertain the role of mitochondria, specifically regulation of synaptic Ca^2+^, in the regulation of synaptic activities (EPSC and IPSC) post rmTBI, tissue sections were pretreated with the protonophore, carbonyl cyanide 4-(trifluoromethoxy) phenylhydrazone (FCCP; 4 μM). FCCP is known to depolarize mitochondria and thereby prevent mitochondrial Ca^2+^ uptake while increasing cytosolic Ca^2+^. Signals were filtered at 2 kHz and sampled at 10 kHz with a Digidata 1440A digitizer and pClamp10 software (Molecular Devices). Series resistance (5–10 MΩ) was monitored before and after the recordings, and data were discarded if the resistance changed by 20%.

### Protocol Design

**FIGURE 1:**
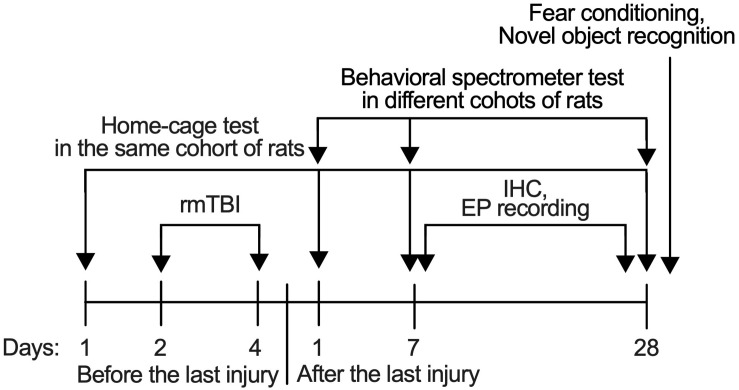
Timeline of procedures. All behavioral tests except home-cage scanning test were performed with different set of rats in different time-points to avoid the influence from another test.

Rats were randomly distributed to have either Sham rmTBI or rmTBI, which was performed 7 days after the rats were received from the vendor. After the injury, they were randomly assigned to groups for behavioral tests, immunohistochemistry, and electrophysiological recordings. Separate groups of rats were used for behavior tests at different time points and with different treatments. Rats exposed to potentially stressful behavioral tests like fear conditioning were not used for further behavioral tests, and also immunohistochemistry staining or electrophysiological recordings (Figure 1). All behavioral tests except home-cage recordings were performed between 9:00 AM to 3:00 PM. The rats were randomly chosen for a given behavioral test during that time period. The sample size was based on statistical power calculation, which was conducted prior to the study. All behavioral tests were performed in a blind manner such that the experimenters performing these tests were unaware of the animal’s treatment. In experiments with immunohistochemistry and electrophysiological recordings, the experimenters were blinded to the different treatments. Some information on the animal study are in the ARRIVE (Animal Research: Reporting of *In Vivo* Experiments) guidelines checklist (Supplementary Document).

### Statistics

Significance testing was performed with Prism 9 (GraphPad Software, La Jolla, CA, United States). A method that combines robust regression and outlier removal (ROUT) in Prism was used to find outliers and those outliers were excluded from further analysis. To compare performance of rats in open field test 1, 7, and 30 days after injury, 2-way analysis of variance (ANOVA) was used first and planned comparison with Bonferroni correction was used if there was no interaction between time factor and treatment factor found. To compare changes from baseline prior to injury in the home-cage system, repeated measures ANOVA with *post hoc* Dunn’s test were used. To compare changes in expressions of GFAP and Iba-1 in 1 week and 1 month after rmTBI, and changes of neuronal activity in 1, 2, and 3 months, 2-way ANOVA with *post hoc* Dunn’s test were used. For the comparison of memory from fear conditioning, non-parametric Friedman repeated measures analysis of variance (ANOVA) with *post hoc* Dunn’s test were used. The time constant (τ) of extinction of cued fear memory in fear conditioning test was measured with a single exponential function of *Y* = (Y0 − Plateau)^∗^exp(−*X*/τ) + Plateau in using Prism 9. To compare changes in neuronal activity 1 week, and 1 month after injury, 2-way ANOVA was used first and planned comparison with Bonferroni correction was used if there was no interaction between time factor and treatment factor. The area under the curve (AUC) showing the effects of the uncoupler, FCCP, on mIPSC/mEPSC during 70-min recording was calculated with the value just before the perfusion of FCCP as baseline from each cell, representing the comprehensive response to the effects of FCCP. Comparison between two groups only were analyzed using t-tests. Significance testing of *P* < 0.05 was considered significant. Data are reported as mean ± standard error of the mean (SEM).

## Results

### rmTBI Did Not Cause Neurological and Motor Deficits

**FIGURE 2:**
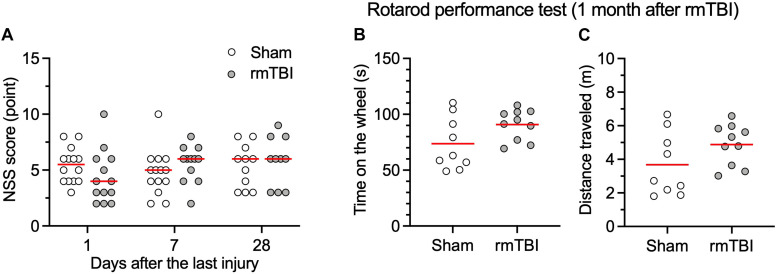
rmTBI on NSS **(A)**, rotarod performance test **(B,C)**. Rats with rmTBI did not show difference in neurological impairment **(A)**, total time on the wheel **(B)**; and total distance traveled **(C)** in the rotarod performance test from rats with sham injury. Black bars in the figure represent mean. Each dot represents data one animal.

In our initial tests, 10 rats subjected to 3J impact resulted in 2 deaths from intracranial hemorrhage and 1 death from nasal hemorrhage, which indicated that this force produces severe TBI. When the impact was reduced to 2J, the survival rate was 100% (*n* = 81) and animals did not show locomotor impairment. We used 2J impact for the rest of the study as a means of reliably producing rmTBI. Neurological impairment after impacts was assessed by using the mNSS 1, 7, and 28 days following injury or sham handling. The results showed no obvious neurological impairment in the days following injury compared to rats with sham control (Figure 2A). To determine whether our mTBI model caused motor disability or motor learning deficits, we performed a Rota-rod test. This also showed no significant difference between injured and sham group (Figures 2B,C). These observations support the characterization of this injury as rmTBI. No outliers were detected.

### rmTBI Did Not Change Exploring Activity of Rats in the Open Field Test

**FIGURE 3:**
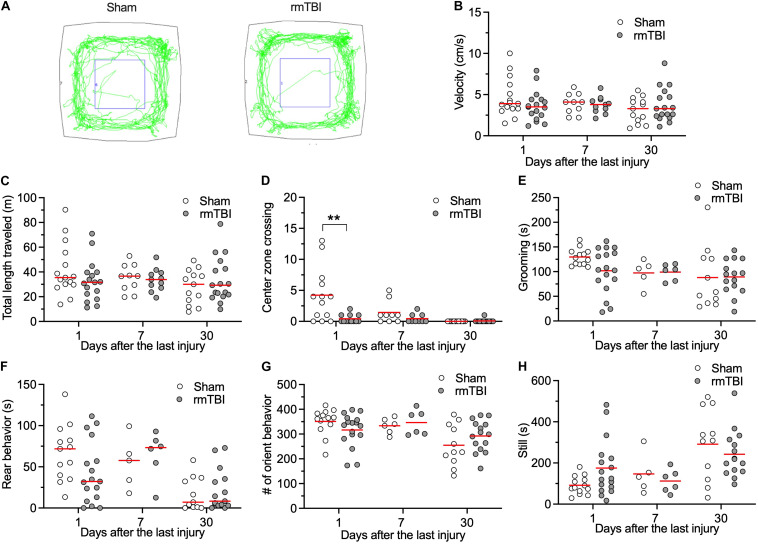
rmTBI on behaviors in Behavioral Spectrometer test. **(A)** Sample tracks recorded from rats with sham injury **(left)** and rmTBI **(right)**. There is no difference in average velocity **(B)**, and total length traveled **(C)** between rats with sham injury and mTBI. Rats with rmTBI show transient reduced intent to cross the middle of the arena **(D)**. There is no difference in time on grooming **(E)**. Rats with rmTBI don’t show changed rear behavior **(F)**, orient behavior **(G)**, and total still time **(H)**. **P < 0.01 by planned comparison with Bonferroni correction. Each dot represents data from one animal.

General locomotor activity level, anxiety, and willingness to explore a new environment was evaluated with the open field test. An automated open field system that combines video and vibration analysis was used for this purpose (Brodkin et al., 2014). rmTBI did not affect general locomotor activity level as determined by the average velocity (Figures 3A,B), and total length traveled (Figure 3C). Rats with rmTBI transiently showed less center zone crossing (Figure 3D, two outlier rats with Sham injury and four outlier rats with rmTBI with high center zone crossing were identified) at day 1 after injury. Rats with rmTBI also did not show changes in other indicators of anxiety: grooming (Figure 3E, one sham outlier rat with low grooming time was identified), rearing behavior (Figure 3F), orienting behavior (Figure 3G), and still time (Figure 3H, one outlier sham rat with high still time was identified). During the test on 5 rats at 7 days after rmTBI, the vibration sensor of the device malfunctioned resulting in some activities not detected (Figures 3E–H). Overall, rats with rmTBI did not show obvious changes in these behaviors detected with the Behavioral Spectrometer.

### rmTBI Acutely Increased Activity of Rats in Their Home-Cages

**FIGURE 4:**
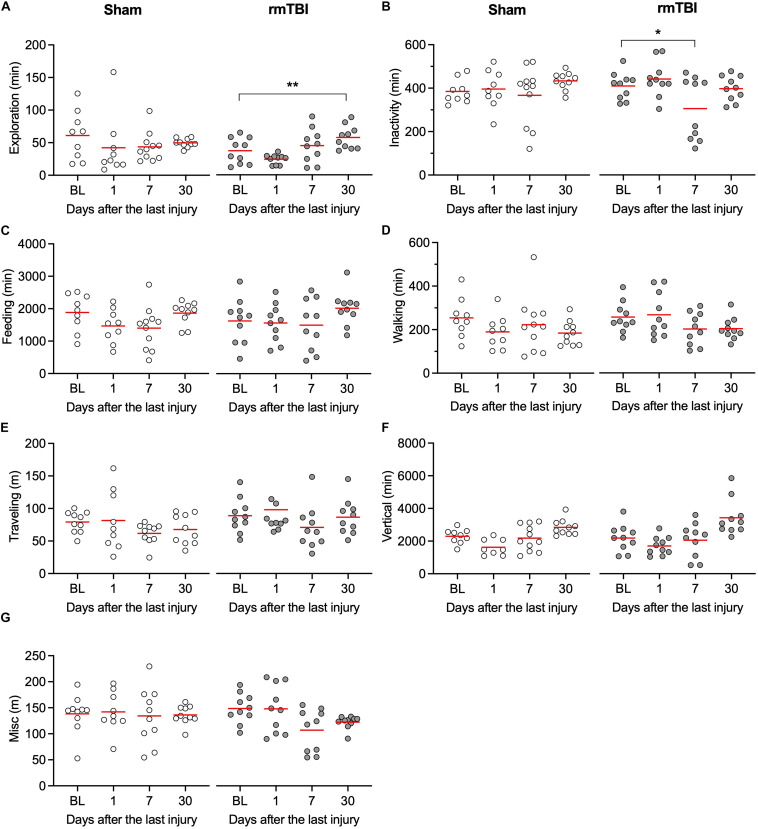
rmTBI on behaviors in home-cage scanning system test. **(A)** Rats with mTBI spend more time on exploration 1 month after injury. **(B)** Rats with injury experience transient reduced activity 7 days after injury. Rats with rmTBI don’t show change in feeding **(C)**, walking **(D)**, traveling **(E)** vertical behavior **(F)**, and all other behaviors **(G)**. *P < 0.05, **P < 0.01 by the Tukey test following one-way repeated-measures ANOVA. Each dot represents data from one animal.

Patients with concussion can experience fatigue, restlessness, and sleeping problems, which are not readily detected in animal models because of extensive experimenter interaction during the behavioral tests. Automated behavioral analysis systems have been developed in the past decade to allow behavioral profiling of animals within their own home cage, providing continuous long-term recording and automated categorization of their spontaneous activities, while minimizing interaction between the animal and the researcher. Rats with rmTBI show increased exploratory behaviors, including burrowing, foraging, and sniffing 30 days after injury (Figure 4A, one sham outlier rat with high exploratory time was identified), and reduced inactivity behaviors 7 days after injury (Figure 4B). However, no significant changes were observed in feeding (Figure 4C), walking (Figure 4D), total length traveled (Figure 4E), or vertical behaviors (Figure 4F). Additionally, no significant changes in body stretching, urinating, and grooming were detected (Figure 4G). Overall, these observations indicate that rats with rmTBI did not show obvious deficits in these spontaneous behaviors in the unstressed home-cage environment.

### rmTBI Caused Long-Term Glial Response

**FIGURE 5:**
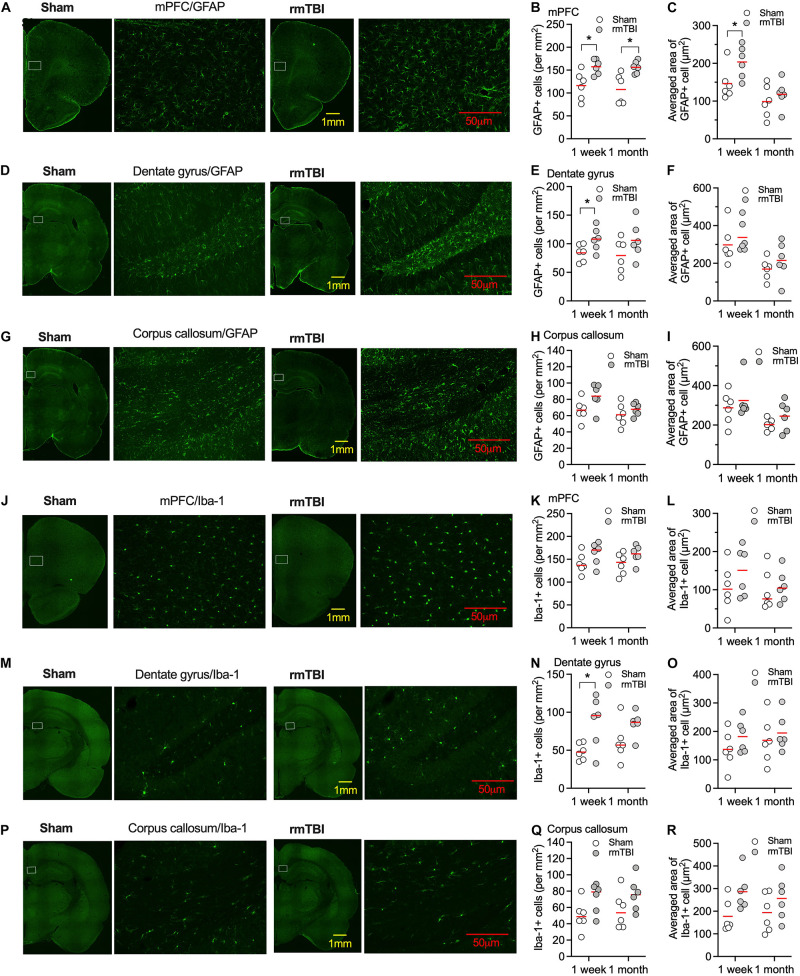
Expression of GFAP and Iba-1 after rmTBI. In mPFC, there are increased number of GFAP+ cells 1 week and 1 month after rmTBI in mPFC **(A,B)**. **(C)** There are increased averaged area of GFAP+ cells 1 week after rmTBI in mPFC. There are increased number of GFAP+ cells 1 week after rmTBI in dentate gyrus **(D,E)**, but not in averaged area of GFAP+ cells **(F)**. **(G–I)** No differences in number of GFAP+ cells and averaged area of GFAP+ cells are found from injury in corpus callosum. **(J–L)** No differences of number of Iba-1+ cells and averaged area of Iba-1+ cells are found from injury in mPFC. **(M–O)** There are increased number of Iba-1+ cells 1 week after rmTBI in dentate gyrus and no differences of averaged area of Iba-1+ cells are found from injury in dentate gyrus. **(P–R)** No differences in number of Iba-1+ cells and averaged area of Iba-1+ cells are found from injury in corpus callosum. White rectangles in images with low power objective magnification represent areas for the images with high power objective magnification on their right side. Each dot represents averaged expression from 3 brain slice sections from each rat. Red bars represent mean. *P < 0.05 by planned comparison with Bonferroni correction.

Elevated microglial and astrocytic responses were observed acutely and sometimes chronically after brain injury (Simon et al., 2017), which might contribute to the poor outcomes of mTBI (Sandhir et al., 2008), or promote brain repair and cognitive functions (Willis et al., 2020). The mPFC, dentate gyrus of the hippocampus, and corpus callosum are the most frequently reported areas relevant to injury after mTBI. We detected changes in microglia and astrocyte density and morphology. Numbers of GFAP positive cells were elevated after 7 days and 1 month post-rmTBI in mPFC (Figures 5A,B). Averaged areas of GFAP positive cells were increased after 7 days, but not 1 month post-rmTBI in mPFC (Figures 5A,C). In the dentate gyrus of the hippocampus, numbers of GFAP positive cells were elevated after 7 days post-rmTBI (Figures 5D,E), but no changes in averaged area of GFAP positive cells (Figures 5D,F). In the corpus callosum, there were no statistically significant differences in the numbers of GFAP positive cells, and averaged areas of GFAP positive cells after rmTBI (Figures 5G–I). Numbers of Iba-1 positive cells were increased 7 days following injury in the dentate gyrus of the hippocampus, but at 1 month post-rmTBI, there were no statistically significant differences in the numbers of Iba-1 positive cells in the mPFC, dentate gyrus, and corpus callosum (Figures 5J–Q). There were no significant differences in averaged areas of Iba-1 positive cells in the brain regions examined after rmTBI (Figures 5J–R). These results indicate transient accentuated glial responses after rmTBI.

Taken together, the results from the behavioral tests and histochemistry staining of astrocyte/microglia indicate that rCHIMERA with 2J energy produced mild and not severe TBI. The results also indicate that our injury model impacted multiple brain regions.

### rmTBI Caused Long-Term Cognitive Deficits

**FIGURE 6:**
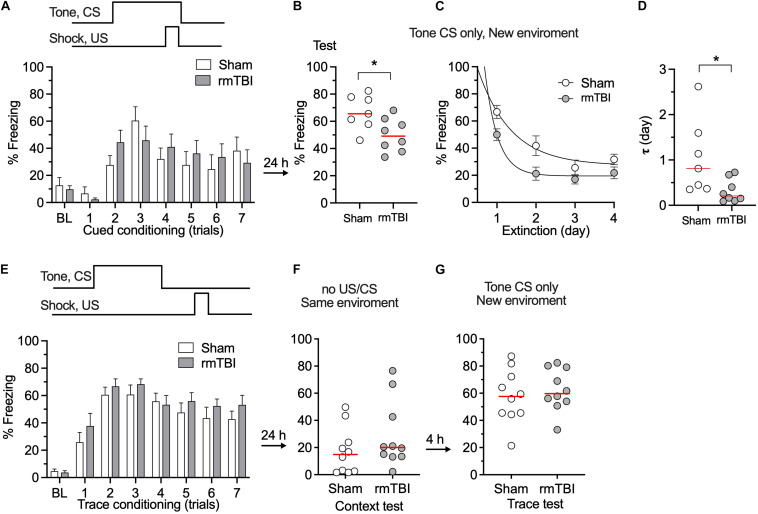
rmTBI on rats in fear conditioning test. **(A)** With cued fear conditioning, rats with sham injury and rmTBI show similar learning. **(B)** Rats with rmTBI show impaired memory in cued fear condition. **(C,D)** Rats with rmTBI show faster extinction of cued fear memory in 4 days test without presence of shock. *P < 0.05 by the *t*-test. With trace fear conditioning, using a delayed unconditional stimulus (US, paw shock) after conditional stimulus (CS, tone), rats with rmTBI and Sham rats show comparable learning **(E)**, and memory **(F,G)**. Each dot represents data from one animal.

**FIGURE 7:**
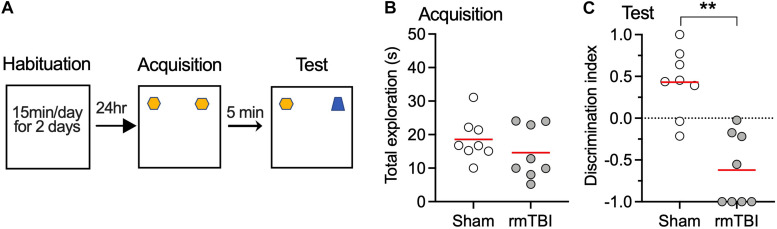
rmTBI on rats in novel object recognition test. **(A)** Scheme of the NOR test. **(B)** Rats with sham injury and rats with mTBI show similar exploration time in training session. Rats with rmTBI show lower discrimination index than sham injury control **(C)** on the novel object in testing session. **P < 0.01 by *t*-test. Each dot represents data from one animal.

To determine whether our rmTBI model replicates the long-term cognitive deficits seen in the clinical setting after rmTBI, fear conditioning test and novel object recognition test were performed 1 month after injury. We used two types of fear conditioning paradigms, delayed conditioning in which the aversive stimulus is presented at the end of a cue (tone), and trace conditioning in which the aversive stimulus is presented with a short interval from the tone (Figure 6A). Following rmTBI, rats did not exhibit different baseline freezing times during fear conditioning training (Figure 6A), which indicates intact fear learning and the expression of freezing after rmTBI. However, the rats exhibited less freezing during the test with tone presentation only (Figure 6B), which indicates reduced cued fear memory after rmTBI. The rats with rmTBI also showed faster extinction of cued fear memory (Figures 6C,D), and no significant difference in trace fear conditioning test with a delayed unconditioned stimulus (foot shock) (Figures 6E–G) was detected. Rats with rmTBI showed a deficit in the novel object recognition test (Figures 7A–C), with reduced discrimination index compared to rats with sham injury (Figure 7C). Taken together, these data show that rats with rmTBI developed long-term cognitive deficits.

### rmTBI Caused Long-Term Abnormal Neuronal Activity in mPFC

**FIGURE 8:**
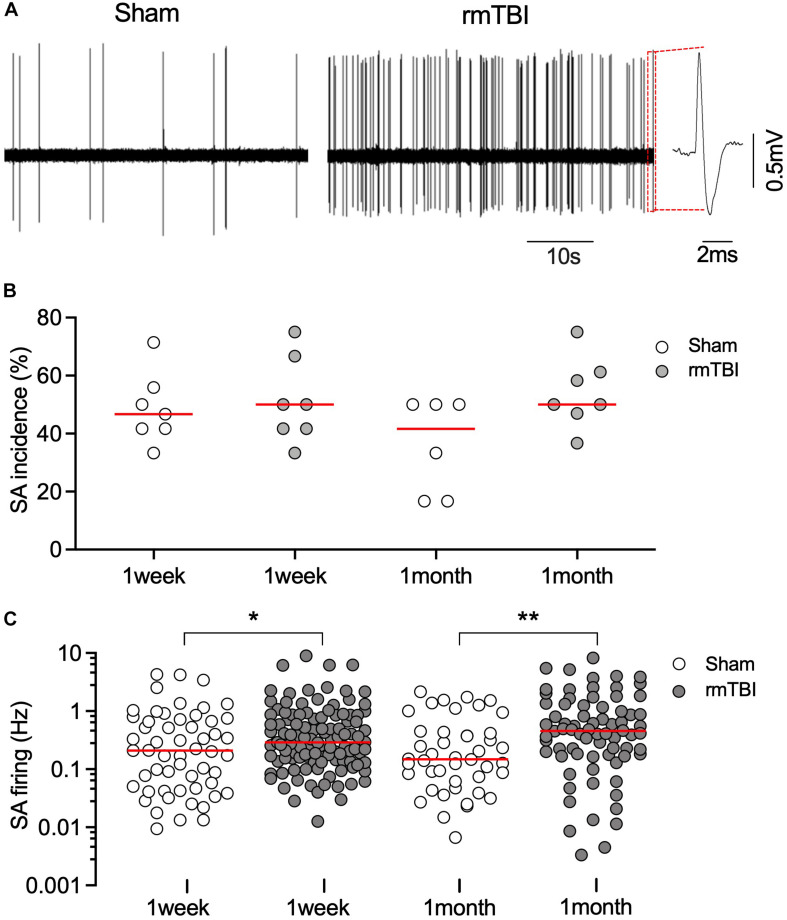
rmTBI on excitatory neuronal activity in mPFC. **(A)** Sample recordings from rat 1 months after sham injury **(left)** and rmTBI **(right)**. **(B)** There is no difference in incidence of spontaneous firing between sham injury and rmTBI groups. Each dot represents recording from one rat. **(C)** Firing rates of spontaneous firing of mPFC pyramidal neurons are increased after rmTBI. Each dot represents data from one neuron recorded (*n* = 6–7 rats per group showing in **B**). Red bars represent means. *P < 0.05, **P < 0.01 by the planned comparison with Bonferroni correction.

mTBI often results in changes in neuronal activity (i.e., abnormal spontaneous action potential generation and synaptic transmission), which contributes to post-traumatic epileptogenesis (Avramescu and Timofeev, 2008; Carron et al., 2016; Ping and Jin, 2016). The mPFC is a critical brain area for avoidance learning and novel object recognition (Barker et al., 2007; Gilmartin et al., 2014), and neuronal activity in the mPFC is relevant to changes in fear conditioning after mTBI (Schneider et al., 2016). In addition, given that there were elevated microglial and astrocytic responses sub-acutely in the mPFC (Figure 5), it is therefore reasonable to postulate that changes in mPFC activity may contribute to the long-term cognitive deficits that follow rmTBI in our model (Figures 6, 7). Using *in vivo* recordings of single unit firing in the mPFC (Figure 8A) of anesthetized rats, we found no significant difference in the incidence of SA between sham and injured rats (Figure 8B). However, there were increased firing rates of recorded units with SA at 1 week, and 1 month after rmTBI compared to age matched sham control (Figure 8C), suggesting long-term increase in spontaneous neuronal activity after rmTBI.

**FIGURE 9:**
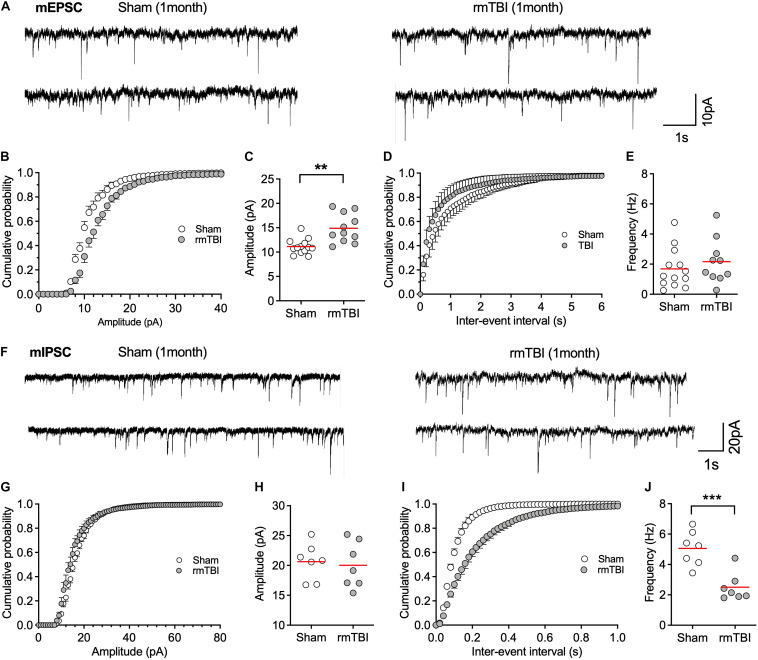
rmTBI on synaptic transmission in mPFC. **(A)** Representative traces of mEPSC from rats 1 month after rmTBI and time-matched control rats with sham injury. **(B)** Cumulative probability and **(C)** average amplitude of mEPSC. **(D)** Cumulative probability and **(E)** average frequency of mEPSC. **(F)** Representative traces of mIPSC from rats 1 month after mTBI and time-matched control rats with sham injury. **(G)** Cumulative probability and **(H)** average amplitude of mIPSC. **(I)** Cumulative probability and **(J)** average frequency of mIPSC. Each dot represents data from one neuron. Recordings were from 6 rats with sham TBI and 6 rats rmTBI. **P < 0.01, ***P < 0.001 by *t*-test.

Shifts in synaptic strength can regulate neuronal activity. The resting level of synaptic events (spontaneous and miniature EPSC or IPSC) reflects transmitter release, the alteration of which may contribute to disordered resting neuronal activity (Zucker, 2005). We found that the amplitude, but not the frequency, of mEPSC was increased 1 month after injury (Figures 9A–E), which represents increased excitatory post-synaptic responsiveness to pre-synaptic glutamate release. In contrast, the frequency, but not amplitude, of mIPSC was markedly decreased 1 month after injury (Figures 9F–J), which represents decreased inhibitory pre-synaptic transmitter release. These together may account for the overall increased postsynaptic neuronal activity observed with single unit recordings (Figure 8).

### Impaired Mitochondrial Function Could Contribute to the Reduced IPSC

**FIGURE 10:**
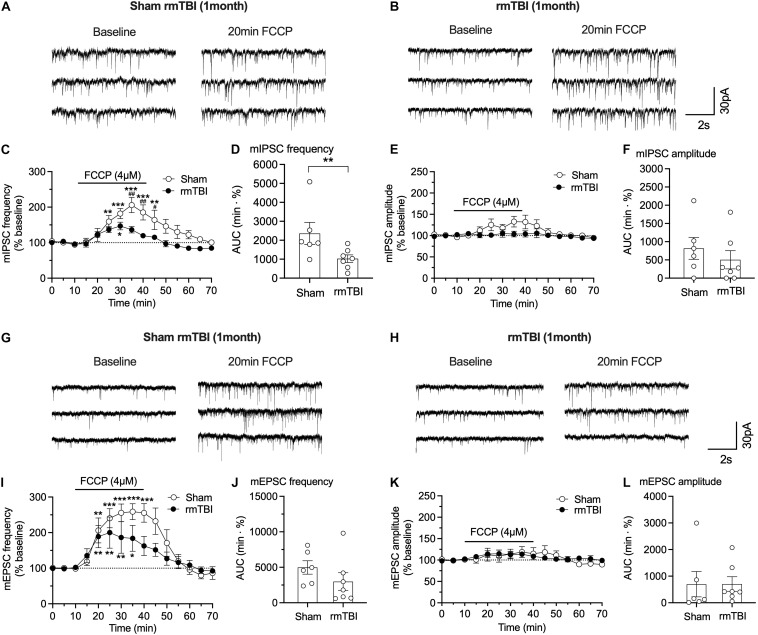
FCCP on synaptic transmission in mPFC after rmTBI. **(A,B)** Representative traces of effects of FCCP on mIPSC from rats 1 month after rmTBI and time-matched sham/control rats. Effects of FCCP on frequency **(C,D)** and amplitude **(E,F)** of mIPSC. **(G,H)** Representative traces of effects of FCCP (4 μM) on mIPSC from rats 1 month after mTBI and time-matched sham/control rats. Effects of FCCP on frequency **(I,J)** and amplitude **(K,L)** of mEPSC. Each dot in **(D,F,J,L)** represents data from one neuron. Recordings were obtained from 8 rats, 4 sham rats, and 4 rmTBI treated rats. *P < 0.05, **P < 0.01, ***P < 0.001 in **(B,F)** were compared to baseline at 0–10 min with 2-way repeated measure ANOVA and Bonferroni correction. ^#^*P* < 0.05, ^##^*P* < 0.01 in **(B,F)** were compared to FCCP perfusion time-matched rmTBI rats with 2-way repeated measure ANOVA and Bonferroni correction. **P < 0.01 in **(C)** was by *t*-test.

Mitochondrial buffering of cytoplasmic Ca^2+^ is important for maintaining normal synaptic transmission; and impaired mitochondrial function in neurological diseases may contribute to impaired presynaptic transmission and subsequent cognitive dysfunctions (Vos et al., 2010; Devine and Kittler, 2018). To test whether the changes in synaptic transmission are attributable to mitochondrial dysfunction, we used the oxidative phosphorylation uncoupler FCCP (4 μM), which can collapse the proton gradient in the inner mitochondrial membrane and thus prevent normal Ca^2+^ uptake and buffering by the organelle. FCCP perfusion increased pre-synaptic GABA release, indicated by the increased frequency of mIPSCs in sham rats during the 30 min perfusion (Figures 10A–C), but the effect of FCCP on the frequency and amplitude of mIPSCs was markedly reduced in rats 1 month after rmTBI compared to sham controls in frequency (Figure 10C) and AUC (Figure 10D). These data indicate impaired mitochondrial function in GABAergic pre-synaptic terminals after rmTBI. In contrast, the amplitude of mIPSCs was not affected by FCCP (4 μM) in either sham rats or rmTBI rats (Figures 10E,F), which indicates that post-synaptic mitochondria did not contribute to the efficacy of post-synaptic GABA receptors to GABA. Like its effects on mIPSC, 30-min perfusion of FCCP (4 μM) produced an increase in frequency of mEPSC in both sham and rmTBI rats (Figures 10G–I), indicating increased pre-synaptic glutamate release. However, the effects of FCCP on mEPSC frequency were not significantly different between sham and rmTBI rats (Figures 10I,J). FCCP (4 μM) did not affect the amplitude of mEPSC in either rmTBI or sham rats (Figures 10K,L).

## Discussion

Cognitive and mood disorders after TBI are major health concerns. However, the mechanisms underlying these phenomena are largely unknown. In this study, we used multiple behavioral tests, detected expression of markers of astrogliosis and microgliosis, examined mPFC neuronal activity, and explored the contribution of mitochondria in changes in synaptic transmission in a CHIMERA-induced rat rmTBI model, with sedation but no anesthesia. Our results show that rats subjected to rmTBI do not show obvious deficits in motor function or spontaneous behaviors (open field, home-cage), but exhibit long-term cognitive impairment, abnormal astrocytic activity, abnormal mPFC neuronal activity, and impaired mitochondrial function. Evidence is also provided that impaired mitochondrial function after rmTBI may contribute to impaired synaptic transmission and subsequent long-term cognitive dysfunction.

Impaired cognitive function contributes to the symptoms of clinical mTBI (McInnes et al., 2017). Fear conditioning and novel object recognition were used in this study to measure cognitive function. Mice with controlled cortical impact injury developed impaired fear recall 7 days after injury (Teutsch et al., 2018). In our study, rats with rmTBI showed reduced fear memory and faster extinction after cued fear conditioning, but impaired memory was not evident in trace fear conditioning, which requires a more intense memory trace (Figure 6). Prefrontal cortex, hippocampus, and amygdala are major brain areas that are involved in learning and memory in fear conditioning, and changes in mPFC activities after mTBI contributes to the impaired fear conditioning (Gilmartin et al., 2014; Schneider et al., 2016). Inactivation of mPFC prior to auditory cued fear conditioning can impair memory for the training context (Gilmartin and Helmstetter, 2010). Combined with our observation that abnormal neuronal activity developed 7 days after the injury (Figure 8), it is possible that changes in fear conditioning test might result from excess mPFC activity after rmTBI. Our finding of impaired novel object recognition (Figure 7) concurs with others who have used it to show memory deficit and impaired recognition memory after mTBI (Morawska et al., 2016; Qubty et al., 2018).

Neuroinflammation is a key feature of TBI (Corps et al., 2015; Simon et al., 2017). Neuroinflammation after TBI can be beneficial as it can promote clearance of tissue debris and tissue regeneration. It can also be potentially harmful, by mediating neuronal death and neurodegeneration (Simon et al., 2017). In TBI, primary mechanical injury to neurons, axons, glia, and blood vessels at the time of trauma results in initial neuronal loss. This is followed by a complex pathogenic cascade including glutamate excitotoxicity, disrupted Ca^2+^ homeostasis, mitochondrial dysfunction, inflammation, free radical generation and lipid peroxidation, apoptosis, and diffuse axonal injury (Xiong et al., 2018). The cause of secondary cell death following TBI might be from neuroinflammation (Hernandez-Ontiveros et al., 2013; Schimmel et al., 2017), involving activation of microglia and astrocytes, and release of inflammatory mediators. However, activated microglia after TBI might be beneficial in the recovery of nerves after injury, because removing microglia from the injury site did not contribute to cognitive dysfunction, and repopulating microglia in affected areas attenuated learning deficits and stimulated neurogenesis (Willis et al., 2020). We detected acute and chronic activation of microglia and astrocytes in multiple brain areas (Figure 5), including the mPFC, consistent with previous reports (Vonder Haar et al., 2019; Willis et al., 2020).

Cognitive impairment, epilepsy, and attention-deficit hyperactivity disorder that follows TBI may be associated with neuronal hyperactivity (Forstner and Knoll, 2020). SA and excitability are transiently depressed for hours after mechanical injury in cultured cortical neurons (Goforth et al., 2011), after fluid percussion TBI (Reeves et al., 2000; Alves et al., 2005), and after controlled cortical impact model of TBI (Ping and Jin, 2016). However, increased neuronal activity was observed 3 days after the TBI and persisted at least for 14 days after the injury (Ping and Jin, 2016). Our model shows long-term increase in neuronal activity after rmTBI (Figure 8) as the result of increased excitatory transmission and reduced inhibitory innervation to mPFC pyramidal neurons (Figure 9), which is consistent with other reports of disrupted glutamatergic and GABAergic synaptic transmission (Almeida-Suhett et al., 2015; McGuire et al., 2019).

Multiple studies indicate mitochondrial dysfunction after mTBI (Wang et al., 2015; Kilbaugh et al., 2016). In a rapid non-impact rotational injury model, mitochondrial respiratory complex I function was reduced in the hippocampus (Kilbaugh et al., 2016). In a rat controlled cortical impact injury model, expression of several mitochondria-associated microRNAs changed after mTBI (Wang et al., 2015). Even though the mechanisms underlying mitochondrial role in TBI are still not clear, a commonly accepted idea is that the rapid membrane stretch during injury causes increased neuronal activity, which in turn increases glutamate release and consequently *N*-methyl-D-aspartate receptor activation, causing elevation of cytosolic and mitochondrial Ca^2+^ levels, resulting in mitochondrial dysfunction (Cheng et al., 2012; Pearn et al., 2017). At the synaptic level, neurotransmitters are rapidly released into the synaptic cleft in response to influx of Ca^2+^ through voltage-gated Ca^2+^ channels, triggered by action potentials. Release and recycling of synaptic vesicles are regulated by Ca^2+^ signaling and are also highly energy-demanding, thereby relying on normal mitochondrial function (Devine and Kittler, 2018).

This is more evident in pre-synaptic terminals where mitochondria are concentrated and are important in regulating neurotransmitter vesicular release matched to energy production and Ca^2+^ buffering (Devine and Kittler, 2018). Mitochondria take up and buffer large amounts of Ca^2+^ from the cytoplasm, which helps shape the cytosolic [Ca^2+^], because of their strong electronegative membrane potential. The uncouplers/protonophore, FCCP, inhibits mitochondria Ca^2+^ uptake by disrupting the proton motive force, via dissipation of the membrane potential, acidification of the matrix and release of buffered Ca^2+^ into the cytosol (Brookes et al., 2004; Camara et al., 2010). Thus, FCCP could increase presynaptic vesicular release in a Ca^2+^-dependent manner (Medler and Gleason, 2002). In this study, we found that changes in pre-synaptic transmission represented by decreased frequency of mIPSC (Figure 10) is, at least in part, attributed to impaired mitochondrial Ca^2+^ handling, possibly including altered buffering capacity after mTBI. Further studies will be needed to delineate the detailed mitochondrial mechanisms involved in regulating excitatory glutamatergic and inhibitory GABAergic synaptic transmission and the role they may play under pathophysiological conditions, for example, rmTBI.

There are some limitations in this study. As an initial study using this injury model, we did not compare effects of impacts with different patterns, e.g., single impact versus multiple impacts with longer intervals to mimic potential scenarios experienced by athletes in sports, veterans in battle field, and elder fallings. Another limitation is that female rats were not used in this study, as male and female adolescent rats following rmTBI showed difference in behavior related to memory and depression, and altered brain structures (Wright et al., 2017). Also, the study on correlation between elevated microglial and astrocytic responses, changes in neuronal activity and mitochondrial function, and behavioral outcomes after rmTBI will be needed.

In summary, we show that rmTBI in rats using the rCHIMERA model, induces: (1) short-term changes in locomotor activity, (2) long-term cognitive impairments, and (3) long-term abnormal cortical neuronal activity and synaptic transmission. These outcomes could in part be ascribed to impaired mitochondrial functions, suggesting that manipulations that modulate mitochondrial functions could represent potential target for interventions to mitigate the long-term symptoms of rmTBI.

## Data Availability Statement

The original contributions generated for this study are included in the article/Supplementary Material, further inquiries can be directed to the corresponding author.

## Ethics Statement

The animal study was reviewed and approved by Institutional Animal Care and Use Committee at the Medical College of Wisconsin.

## Author Contributions

YF and DC: performing the experiments and analyses. KL and ER: performing the experiments. CM: analysis and preparing figures. QH: design, writing, and editing. CP: design. W-MK and AC: design, review, and editing. BP: design, writing, editing, and analyses. All authors contributed to the article and approved the submitted version.

## Conflict of Interest

The authors declare that the research was conducted in the absence of any commercial or financial relationships that could be construed as a potential conflict of interest.

## Publisher’s Note

All claims expressed in this article are solely those of the authors and do not necessarily represent those of their affiliated organizations, or those of the publisher, the editors and the reviewers. Any product that may be evaluated in this article, or claim that may be made by its manufacturer, is not guaranteed or endorsed by the publisher.

## Footnotes

1
https://imagej.nih.gov/ij/
